# Influence of Ceramic Particles Size and Ratio on Surface—Volume Features of the Naturally Derived HA-Reinforced Filaments for Biomedical Applications

**DOI:** 10.3390/jfb13040199

**Published:** 2022-10-21

**Authors:** Aura-Cătălina Mocanu, Florin Miculescu, Cătălina-Andreea Dascălu, Ștefan Ioan Voicu, Mădălina-Andreea Pandele, Robert-Cătălin Ciocoiu, Dan Batalu, Sorina Dondea, Valentina Mitran, Lucian-Toma Ciocan

**Affiliations:** 1 Department of Metallic Materials Science, Physical Metallurgy, Faculty of Materials Science and Engineering, University Politehnica of Bucharest, 313 Splaiul Independentei, J Building, 060042 Bucharest, Romania; 2 Department of Analytical Chemistry and Environmental Engineering, Faculty of Applied Chemistry and Materials Science, University Politehnica of Bucharest, 1-7 Gh. Polizu Str., 011061 Bucharest, Romania; 3 Advanced Polymer Materials Group, University Politehnica of Bucharest, 1-7 Gh. Polizu Str., 011061 Bucharest, Romania; 4 Department of Biochemistry and Molecular Biology, University of Bucharest, 91-95 Spl. Independentei, 050095 Bucharest, Romania; 5 Prosthetics Technology and Dental Materials Department, “Carol Davila” University of Medicine and Pharmacy, 37 Dionisie Lupu Street., 020022 Bucharest, Romania

**Keywords:** ceramic-reinforced filaments, printable filaments, particle size, ratio influence

## Abstract

The intersection of the bone tissue reconstruction and additive manufacturing fields promoted the advancement to a prerequisite and new feedstock resource for high-performance bone-like-scaffolds manufacturing. In this paper, the proposed strategy was directed toward the use of bovine-bone-derived hydroxyapatite (HA) for surface properties enhancement and mechanical features reinforcement of the poly(lactic acid) matrix for composite filaments extrusion. The involvement of completely naturally derived materials in the technological process was based on factors such as sustainability, low cost, and a facile and green synthesis route. After the HA isolation and extraction from bovine bones by thermal processing, milling, and sorting, two dependent parameters—the HA particles size (<40 μm, <100 μm, and >125 μm) and ratio (0–50% with increments of 10%)—were simultaneously modulated for the first time during the incorporation into the polymeric matrix. The resulting melt mixtures were divided for cast pellets and extruded filaments development. Based on the obtained samples, the study was further designed to examine several key features by complementary surface–volume characterization techniques. Hence, the scanning electron microscopy and micro-CT results for all specimens revealed a uniform and homogenous dispersion of HA particles and an adequate adhesion at the ceramic/polymer interface, without outline pores, sustained by the shape and surface features of the synthesized ceramic particles. Moreover, an enhanced wettability (contact angle in the ~70−21° range) and gradual mechanical takeover were indicated once the HA ratio increased, independent of the particles size, which confirmed the benefits and feasibility of evenly blending the natural ceramic/polymeric components. The results correlation led to the selection of optimal technological parameters for the synthesis of adequate composite filaments destined for future additive manufacturing and biomedical applications.

## 1. Introduction

The modern manufacturing industry is facing increasingly more challenges when it comes to biomedical products that need to be provided with specific requirements because of the application field and usually in a short, timely manner. Hence, additive manufacturing (AM), also known as 3D printing, has become a hotspot among the 21st century manufacturing technologies, mostly due to the high accuracy, great aesthetics, and low cost that it can inflect upon a wide range of tailor-made products [1,2].

Given the current socioeconomic advances, the efforts in most engineering lines are guided toward the development of renewable feedstocks, including for the biomaterials field [3,4,5,6]. Specifically, it was reported that with the rapid aging of the population and the irreversible effect of damaged bones due to the related osteoporosis, tumor resections, and congenital defects or accident-induced trauma, the number of medical interventions for tissue restoration is spiraling annually [2,7,8,9,10].

On this account, researchers have sought to manufacture different structures for bone tissue reconstruction based on either metallic, polymeric, ceramic, or any suitable combination between these biocompatible materials, in order to recreate the natural composite architecture [11,12,13,14,15]. The biogenic angle was also targeted for the ceramic ones, especially the calcium phosphates (CaPs) division (e.g., hydroxyapatite (HA), tricalcium phosphate (TCP), and biphasic HA/TCP) for which several bioresources have been identified as compatible precursors for facile and reproducible synthesis methods (e.g., bovine, sheep or fish bones [12,16,17], and marble or marine seashells [4,18,19]). The interest stirred up by the naturally derived calcium phosphates, mostly HA, is due to its high resemblance with the inorganic matter of the natural bones (e.g., chemical composition, structural features, biocompatibility, and osteoconductivity) [4,11,16,20].

Moreover, the type and ratio of the polymeric component (i.e., organic matter) are also of crucial importance when involved for bone-like composite structures development [21]. This translates to the application of AM techniques to a restricted range of polymers. For example, the most popular method used in orthopedics, Fused Deposition Modeling (FDM)/Fused Filler Fabrication (FFF), requires typical materials with thermoplastic properties (e.g., poly(lactic acid) (PLA), poly(ε-caprolactone) (PCL), thermoplastic polyurethane (TPU), and acrylonitrile butadiene styrene (ABS)) necessary for extrusion in the form of filaments at specific temperatures through different-sized nozzles [2,7,22,23,24]. Among them, only PLA is of natural origin (derived from maize, beet, or sugarcane) and is also approved by the Food and Drug Administration as biocompatible, biodegradable, nontoxic, and overall patient-risk-free [3,8,25]. As compared to the medicinal PLA, the one used in AM presents distinctive lower molecular weight and flexibility for facile extrusion [26].

However, the use of polymeric components alone proved challenging for bone structures development due to unsuitable mechanical (e.g., brittleness) and surface abilities (e.g., poor adhesion for cells or proteins attachment) for the final application [20,21,27,28,29,30]. Similarly, structures consisting of only HA indicated a slow degradation rate and a reduced porosity level several months after implantation [23,31]. As such, recent dedicated studies focused on the necessity to create advanced materials, namely advanced composites based on commercial/synthetic CaPs and a polymeric matrix (mostly PLA) [7,20,24,26,29,30,32,33,34]. When prospecting the synthesis of CaPs/PLA filaments as promising feedstock materials for the FDM method, several issues were raised: (i) the poor interfacial adhesion given by the opposed wettability behavior of the two components, (ii) the optimum CaPs ratio in order to ensure a uniform and homogenous dispersion into the polymer matrix, (iii) the appearance of subsequent porosity at the particle–matrix interface, (iv) the adequate overall hydrophilic character of the mixtures, and (v) the mechanical takeover function of CaPs ratio and dispersion degree.

Several chemical and physical methods for surface modification were deployed and reported in order to improve the interplay between the two materials (e.g., addition of binders, polyacids/acrylic acids, plasticizers, and impact modifiers), yet not always with the most desired results [7,25,27,29,30]. Moreover, the ratio of admixed CaPs varied in all studies from 3 wt.% to a maximum of 20–40 wt.% with different increments, and led to either a random distribution or agglomeration of the particles, or both [20,26,30,32,34]. A higher ratio of CaPs (>20 wt.%) was also associated with the deformation of the extruded filaments in terms of diameter size and surface regularity [26,29,34]. Nonetheless, the higher the content of CaPs, the higher the regenerative ability of the composite structure, due to the formation of more nucleation sites for apatite species deposition [25,34]. Hence, the CaPs ratio was stretched in a few studies to the maximum of 50 wt.% [24]. Coupled with the (micro)spherical shape of the particles, all sequences with high CaPs loading induced a supplementary porosity at the interface with the polymeric matrix and reduced the mechanical strength values implicitly [24,29,33]. However, starting from the lowest ratios of CaPs, the wettability behavior improved significantly, leading to progressively reduced contact angles and prospective cell attachment/adhesion when compared to the polymer alone [20,21]. Despite all the strategies engineered for composite filaments development, no study has revealed a clear examination and delimitation for the optimum ratio, size, or shape of the CaPs particles loaded into the polymeric network.

Hence, we propose the incorporation of naturally derived CaPs (i.e., bovine bone HA) into the polymeric matrix (i.e., PLA), without binders or surface modifiers added during preparation and engineering of a completely natural composite filament type destined for scaffolds manufacturing with biomedical applicability. To the best of our knowledge, only one study has reported the addition of natural-fishbone-derived HA into the PLA matrix, but it lacks substantial information in regard to the processing technique, investigation stages, and results for the obtained materials [31].

In order to solve the abovementioned issues and address all particular requirements for ceramic/polymeric filaments, this study provides for the first time the concomitant modulation of two HA-specific parameters: the incorporated ratio (five increments of 10% each) and the particles size (three-dimensional ranges), always leading to a uniform dispersion into the polymeric matrix. More to the point, a subsequent novel element of our strategy relies on the HA particles shape—polyhedral geometry with sharp and rounded edges—which has not been reported anywhere in the literature. The particles shape along with their microporous surface constitute inherent factors of the HA isolation and extraction technology from bovine bone sources [12,17,35], both favorable for the elimination of outline pores at the HA/PLA interface and for the proper/strong adhesion of the two components. Our strategy leads, therefore, to a tunable synthesis technology for naturally derived composite filaments with precise optimal size and ratio of the bovine bone HA particles highlighted through a complex surface → volume investigation program comprised of morpho-compositional, wettability, micro-CT, and compressive strength analyses. Furthermore, the results offer a complete view over the benefits of natural HA acting also as mechanical reinforcement for PLA-based filaments.

## 2. Materials and Methods

### 2.1. Samples Preparation

The initial natural colored PLA materials were purchased from local suppliers (Merck KGaA, Darmstadt, Germany) and were involved in the proposed investigation program without any chemical or physical treatment. The diameter of the PLA granules specified by the manufacturer is 2 ± 0.5 mm.

The ceramic material was synthesized by conversion of bovine bones into natural hydroxyapatite (HA) by an already established, completely reproducible, and previously reported procedure (based on repeated boiling in distilled water and three successive thermal treatments applied up to 1200 °C) in detail in Refs. [16,17,35]. After ball mill grounding (450 rpm/2 h) and granulometric sorting of the HA powder with standardized sieves—meshes of 200 μm → 40 μm (Retsch GmbH, Haan, Germany) [36]—three size sorts were chosen for the addressed objective: <40 μm, <100 μm, and >125 μm. Moreover, based on the already published data, the involved HA is nanocrystalline, monophasic, and without any traces of other compounds, even at high temperatures [16,17].

The fabrication of the composite filaments involved the incorporation of different ratios of HA, from each size sort, into the PLA matrix, in the 0–50% range, with an increment of 10%. Hence, the samples were denominated according to the granulometric HA sort and the afferent PLA ratio (e.g., *100% PLA, 90% PLA, 80% PLA, 70% PLA, 60% PLA,* ad *50% PLA*). The 100% PLA samples were considered the control/reference materials.

For each sort of HA powder, and each HA/PLA ratio, the process required the following steps: (1) the mechanical homogenization of the two materials was carried out at 50 rpm for 1 h in a tumbler mixer (Inversina, Bioengineering AG, Zürich, Switzerland), and (2) the resulting mixtures deposited in sterile glass dishes were further subjected to thermal homogenization by continuous stirring on the magnetic stirrer hob, up to the melting stage, at constant temperature (190 °C). Half of the obtained slurry, from each sample type, was converted into cylindrical pellets by casting and compression into cylindrical molds (*cast mixtures*: Φ = 14 mm, h = 16 mm). The cast mixtures pellets were turned to the same diameter and height and the plane-parallel surfaces were acquired by grinding on abrasive paper (P600–2500). The other half of the slurry was let to cure/cool and harden and then the slats/films of mixtures were cut into small strips as to facilitate the final grounding process.

The obtained grounded mixtures (small-sized granules) from each sample type were further used for the extrusion of the composite filaments with uniform distribution of the ceramic particles into the polymeric matrix (Pro Filament Extruder, Noztek, West Sussex, UK). The filaments were formed at 200 °C and 10–15 rpm, because of the increased HA ratio. After exiting the extruder nozzle (1.2 mm in diameter), they were air-cooled and coiled on a spool.

For a proper evaluation of their features, the internal surfaces of cast mixtures and extruded filaments were exposed by cross-sectioning with a diamond disk and grinding on abrasive paper (P600–2500).

### 2.2. Samples Characterization

(a) The macro- and microstructure of the samples, in both surface and cross-sectional view, were evaluated by scanning electron microscopy (SEM), using a Phillips XL 30 ESEM TMP microscope (FEI/Phillips, Hillsboro, OR, USA) coupled with an auxiliary microanalysis EDS system (EDAX Sapphire UTW, 128 eV resolution). The acquisition of micrographs after casting and extrusion was conducted on 5 randomly selected areas. The HA particles size was inferred by processing the SEM micrographs with the help of ImageJ software (National Institutes of Health, USA).
(b) The differential scanning calorimetry (DSC) curves were acquired using Netzsch DSC 204 F1 Phoenix equipment (Netzsch, Selb, Germany). The samples were heated from room temperature (RT) up to 300 °C, at a heating rate of 5 °C/min in air ambient.
  The degree of crystallinity (X_c_, %) was computed according to Equation (1) where $\Delta H_{c}$ is the crystallization enthalpy and $\Delta H_{m}$ is the melting enthalpy of the sample. The ideal melting heat of 100% crystalline PLA ($\Delta H_{m}^{0})$ was considered as 93.7 J [29,37,38,39].
  (1) $$X_{c}=\frac{\Delta H_{m}-\Delta H_{c}}{\Delta H_{m}^{0}}\times 100$$
(c) The wettability was evaluated by water contact angle measurements using a Krüss Drop Shape Analyzer—DSA100 (A. Krüss Optronic GmbH, Hamburg, Germany). The experiments were performed with three wetting agents (water, diiodomethane (DIM), and ethylene glycol (EG)) at 20 ± 1 °C and room humidity of 45 ± 5%. The images were captured 1 s after the wetting agent droplet deposition. The results (average of 5 determinations/sample) were then processed with the ImageJ 1.50 software (National Institutes of Health, USA). The surface free energy was calculated by the Owens, Wendt, Rabel, and Kaelble (OWRK) method [40].
(d) The micro-CT analysis of the extruded filaments was conducted on a Brucker micro-CT SkyScaner 1272 model. The products were cut into specimens with ~4 mm length from the middle of each sample/filament wire. The scanning was performed without any filter, at a voltage of 50 kV, a current of 175 µA, a rotation set of 0.2°, and 4 average frames per capture. Each sample was rotated to 360°. Images were processed using a NRecon 1.7.1.6 reconstruction software from Bruker micro-CT.
(e) The compression test was performed on the cast mixtures pellets using a universal test machine Walter + Bai AG, Loehningen (Schaffhausen, Switzerland), type LFV300. The used test speed was 1 mm/min with an acquisition rate of 0.01 s. The shortening and barreling deformation degrees were calculated by measuring the cast mixtures pellets (diameter and height) before and after performing the compression tests. The results represent the average of three sets of measurements/sample type.

## 3. Results and Discussion

### 3.1. Morpho-Compositional Evaluation

**Figure 1:**
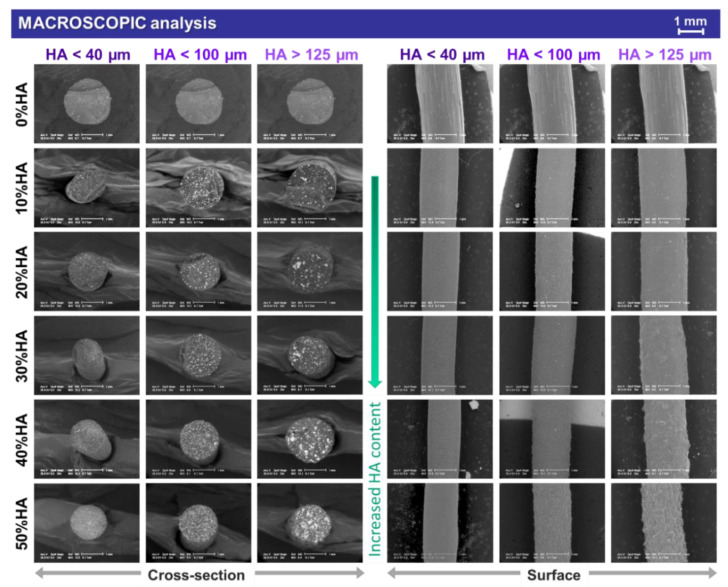
Representative macroscopic images on cross-section and surface of the extruded filaments obtained from PLA and three-dimensional sorts of HA powder with 0–50% ratio.

The macrostructure of all extruded composite filaments reinforced with bovine bone-derived HA with different particle sizes and ratios is presented in Figure 1. In addition, supplementary information is provided in Figure S1 for HA particle size analysis and the results concur with those previously reported in Refs. [16,17]. The assessment of the top-view (surface) and cross-sectional surface view of the samples was performed in order to reveal the dispersion degree of ceramic particles into the polymeric volume and the influence of both the ceramic particles size and ratio on the filaments integrity and full-length uniformity.

The PLA-based samples (also used as reference) revealed a smooth surface topography preserved on the entire length of the filaments. Starting with small-sized HA particles (<40 μm) incorporated in the polymeric matrix, the influence of the ceramic ratio became visible only at high loading concentration (50%), when slight irregularities could be spotted but only in top-view (surface) mode. The roughness effect was more pronounced once the HA particles size increased, leading to the appearance of micrometric and round protuberances on the filaments surface. They can be depicted in both top-view and cross-sectional surface view starting from a 20% HA ratio. When the maximum HA particles size (>125 μm) and ratio (50%) were reached, no smooth area could be obtained on the filaments surface.

For all cross-sectioned samples, at all HA ratios, the uniform dispersion degree of the ceramic counterpart in the polymeric volume stood as an indicator of the homogenous synthesized mixtures, independent of the HA particles size. Moreover, the extrusion process led to no preferential arrangement of the ceramic particles inside the filament, with few particles reaching the outer shell, regardless of the dimensional range.

**Figure 2:**
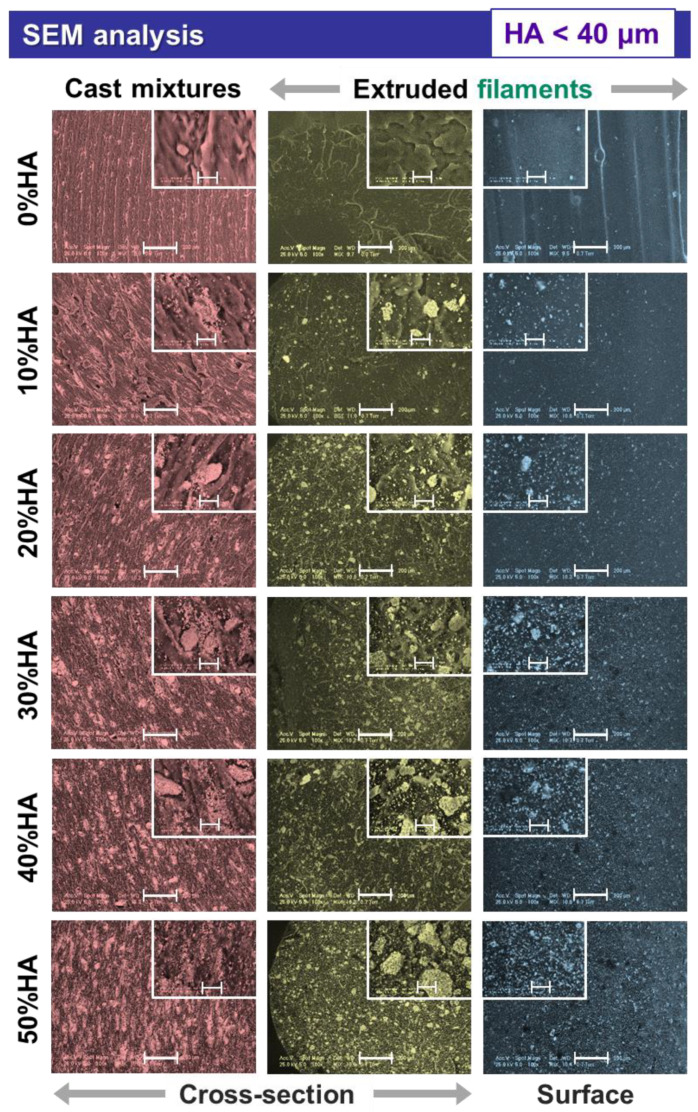
Morphological characterization of the cast mixtures and the extruded filaments obtained from PLA and HA powder (particles < 40 μm) with 0–50% ratio. Scale bar: main image—200 μm, inset—20 μm.

**Figure 3:**
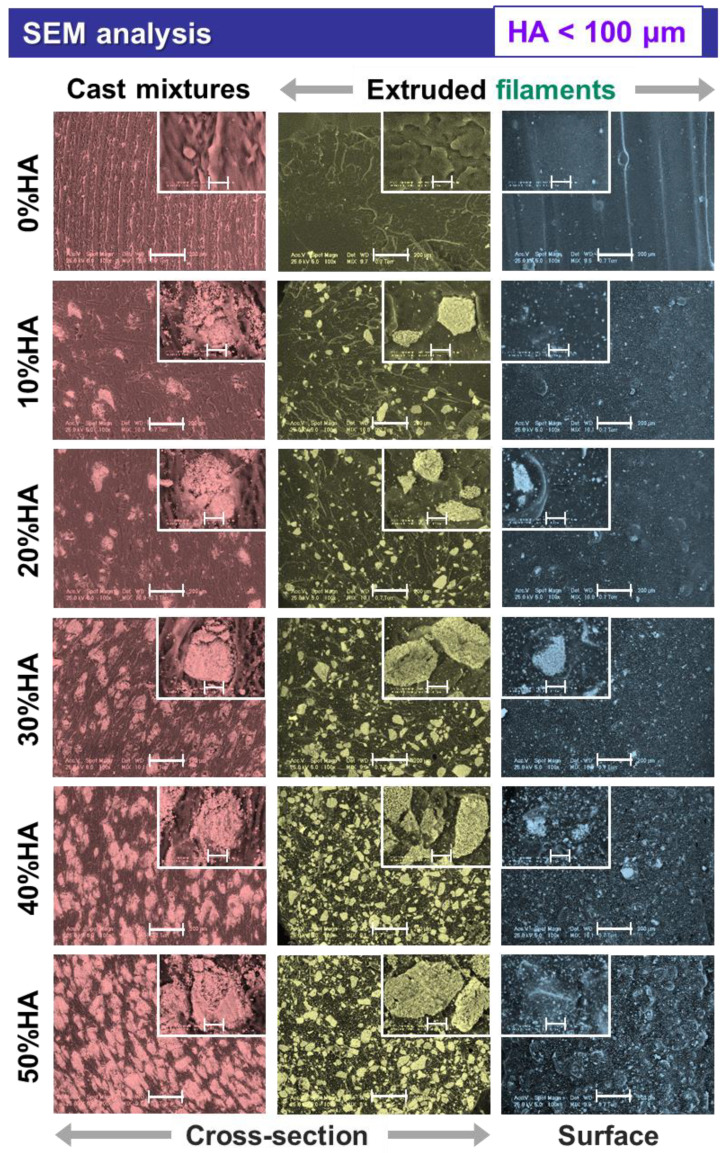
Morphological characterization of the cast mixtures and the extruded filaments obtained from PLA and HA powder (particles < 100 μm) with 0–50% ratio. Scale bar: main image—200 μm, inset—20 μm.

**Figure 4:**
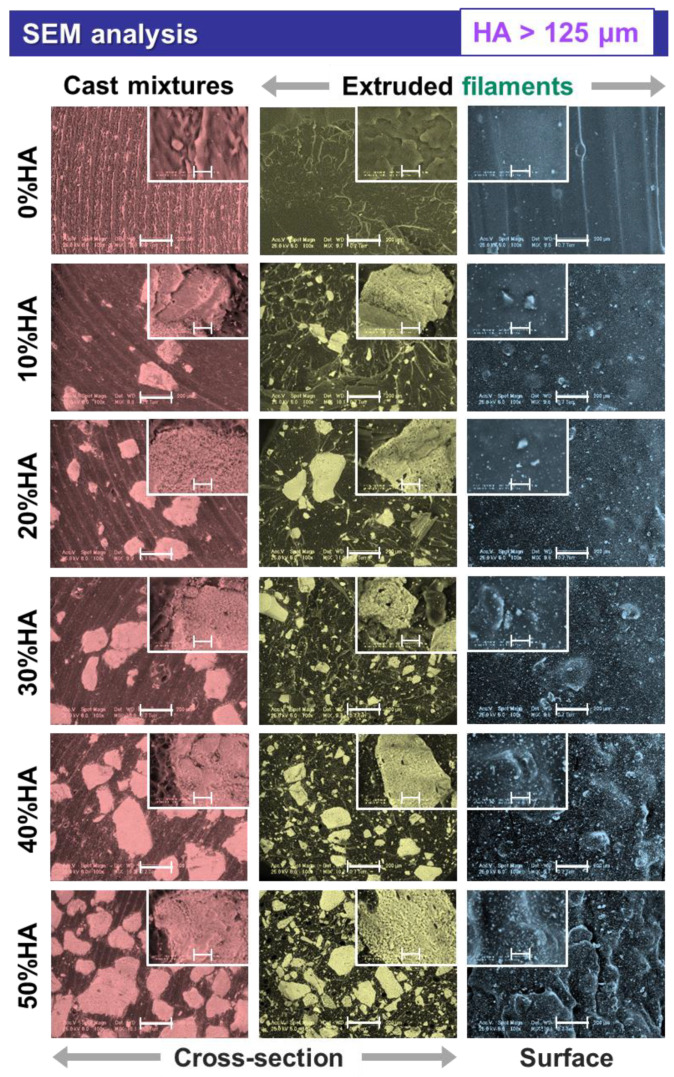
Morphological characterization of the cast mixtures and the extruded filaments obtained from PLA and HA powder (particles > 125 μm) with 0–50% ratio. Scale bar: main image—200 μm, inset—20 μm.

When analyzed at higher magnification, the micrographs exposed supplemental features for the extruded filaments and also the cast mixtures pellets, as presented in Figure 2, Figure 3 and Figure 4, afferent for each HA dimensional range.

In the case of all extruded filaments, based on the uniform particle distribution incremented with the increase in the HA ratio, the smallest-sized particles filled the space between the larger ones, leading to a gradually maximized surface coverage as seen from the cross-sectional view. As compared to the cross-sectional view of the cast mixtures pellets, the polyhedral shape and coarse surface of the particles were preserved for all three-dimensional ranges. However, during the extrusion process, the particles edges were converted from sharp to round and no aggregates were formed. By casting the composite HA/PLA mixtures, the ceramic particles tended to conglomerate once the ratio was increased (>30%), and the distribution was scarcely uniform at higher particle sizes (<100 μm and >125 μm) (Figure 3 and Figure 4), as compared to the extruded filaments for which the HA dispersion was uniform.

Another important feature depicted from the cross-sectional view of the extruded filaments was the absence of free spaces or circular pores at the ceramic particle–polymeric material interface, regardless of the particles sizes. Moreover, as a result of the HA synthesis route, the particles surface was covered by small pores [12], generating a favorable microporosity. Thus, the higher specific surface area of the ceramic particles ensures a facile adhesion to the PLA polymeric surface; this has often been challenging to achieve without the involvement of additional chemical modifiers during the preparation of commercial HA/PLA composites [29].

The addition of incremented higher HA ratios led also to a proper packing and enclosing of the ceramic particles in the polymeric network. Therefore, the involved naturally derived HA acted as a reinforcing component. Previous studies conducted with (micro)spheres of commercial HA reported that it acts as an adjuvant factor for porosity generation inside the filament, rather than as a reinforcement material [33].

Further, the surface-top-view of the filaments suggest that smoother surfaces, similar to those of the raw PLA ones, can be obtained only by incorporating the smallest HA particles (Figure 2). After adding the larger particles, variable micrometric bumps started to appear on the filaments surface in an irregular manner. Once the HA ratio was increased (>20%), the numerous overlapped bumps formed significant protuberances (clearly seen at 50% HA), which could lead to some issues when printing the filaments (e.g., clogging/accumulation of HA particles or composite mixture in the printer nozzle [20,22]) and lower quality of the final products [34]. Therefore, for a favorable surface modification of PLA [27,30], the proper filaments dimensions for 3D printing could be achieved by modulating the nozzle diameter, extrusion velocity, temperature, and filling [41] according to the HA particles size and ratio.

**Figure 5:**
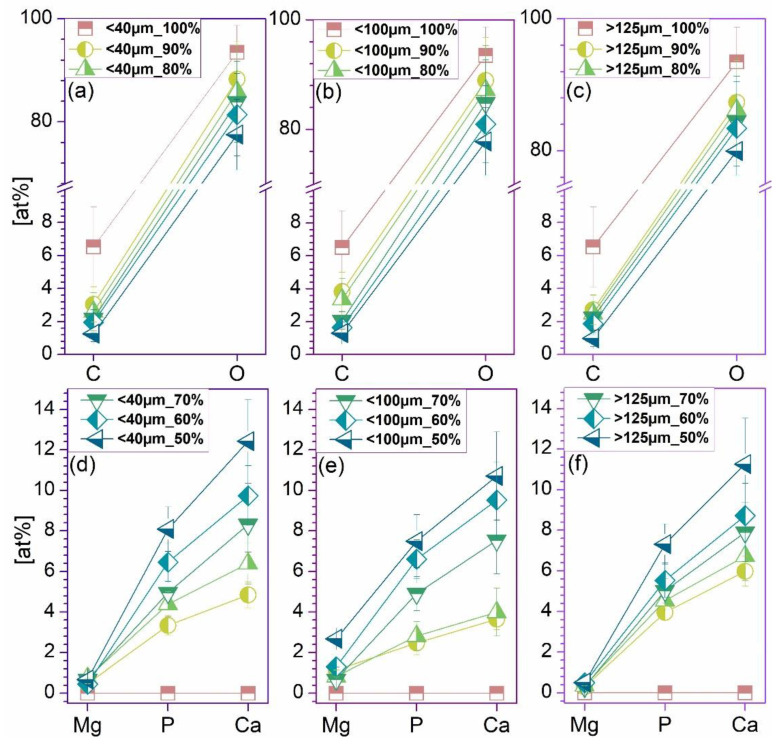
Compositional evaluation of the extruded filaments obtained from PLA and three-dimensional sorts of HA powder (<40 μm—(**a**,**d**), <100 μm—(**b**,**e**), and >125 μm—(**c**,**f**)), each with 0–50% HA ratio.

The comparative EDS analysis performed on all extruded filaments in cross-sectional view revealed the characteristic composition of calcium phosphate/polymeric materials based on C, O, Ca, and P elements (Figure 5). In addition, minor amounts of Mg were detected for the 10–50% addition of ceramic particles, which was expected for the specific bovine-bone-derived HA [16]. Further, independent of the particles dimensional ranges, the amount of carbon dropped drastically once the HA ratio was incremented and, implicitly, a higher degree of coverage was assured (Figure 2, Figure 3 and Figure 4). Similarly, the oxygen concentration presented a decreasing trendline with only a few percent, but in this case, the reduction was proportional with the increase in the HA ratio. Regarding the calcium, phosphor, and magnesium elements, their variable concentrations spiked in an upward trendline with the incremented addition of HA. These minor differences can be assigned to the varying surface roughness, particles sizes, or the overall method inaccuracies [42,43,44].

### 3.2. Differential Scanning Calorimetry (DSC)

**Figure 6:**
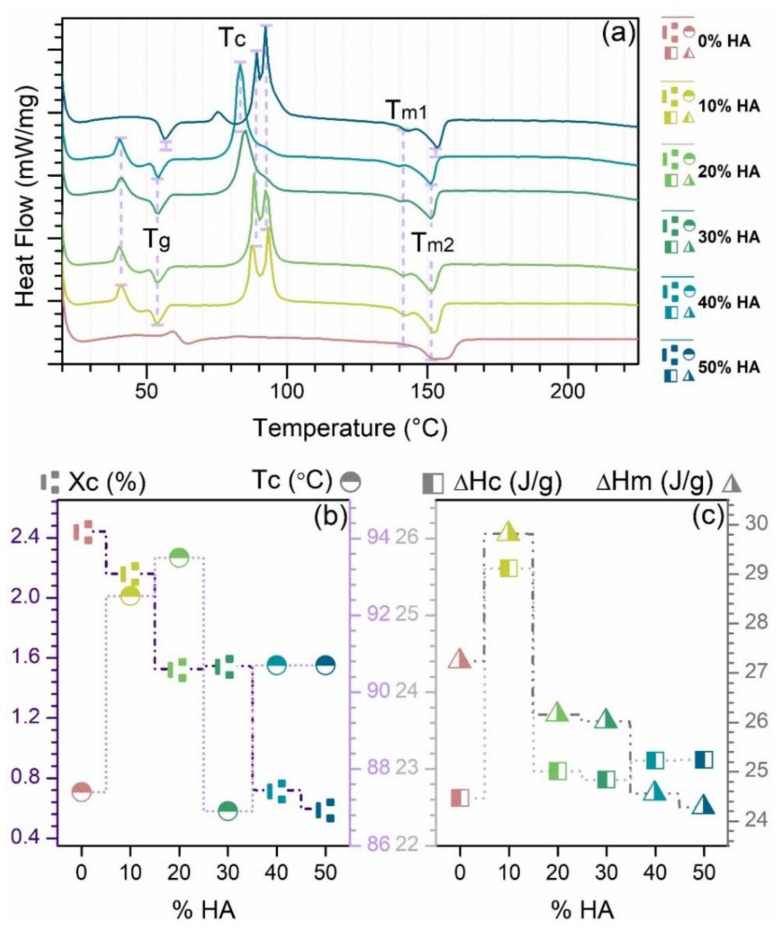
(**a**) DSC curves, (**b**) evolution of the degree of crystallinity (Xc) and the temperature of crystallization (Tc), and (**c**) evolution of the crystallization enthalpy (∆H_c) and the melting enthalpy (∆H_m) of the extruded filaments obtained from PLA and HA powder with 0–50% ratio.

The thermal properties of the obtained composite filaments with 0–50% HA ratio were investigated by DSC analysis, plotted in the 20–225 °C range, and the results are shown in Figure 6. According to the DSC curves (Figure 6a), all samples exposed two endothermic peaks at approx. 140–142 °C and 151 °C due to the progressive melting of PLA [37,45], independent of the HA ratio. However, the incorporation of HA particles led to the appearance of an exothermic peak specific for each sample in the ~86–93 °C range (at lower and maximum HA ratios, it arose tip-split) and corresponding to the polymer crystallinity—cold crystallization (temperature of crystallization, T_c_). According to the T_c_ evolution, its downward trend once the HA ratio increased is related to the heterogeneous crystallization of samples with more HA particles, which can act as nucleating agents for PLA crystals [37,39].

Moreover, all composite samples showed another endothermic peak indicative of the glass transition temperature (T_g_). The T_g_ development was found to increase with the gradual increment in HA ratio from ~53 °C to ~57 °C. This phenomenon was previously centered and explained based on the influence of the HA particles amount per unit volume in the composite, and it was concluded that the T_g_ is dependent on the afferent raised surface area of the HA particles [45]. Additionally, an increase in the interfacial area caused by a higher loading concentration of HA particles enhanced the flexibility of the polymer chain, thus lowering the T_g_ [29,46].

The degree of crystallinity (Figure 6b), calculated based on the values presented in Figure 6c (for the crystallization and melting enthalpies), ranged from 2.44 (100%PLA) to 0.6 for samples with incorporated HA (50%). By advancing the HA ratio, the X_c_ values decreased, and as such, the orientation and mobility of the polymer chains/macromolecules was hindered by a higher degree of disorder [6,29,45,46]. Therefore, one can conclude that due to the HA particles tendency to form nucleation centers in the polymeric matrix, more rigid phases are modeled and govern further the final features of the products [47], as also seen in Figure 1, Figure 2, Figure 3, Figure 4 and Figure 5.

### 3.3. Contact Angle and Surface Energy Investigations

An important feature of the HA/PLA mixtures surface for an adequate future biological response is the wettability/hydrophilicity and surface free energy (SFE). In general, cell adhesion, proliferation, migration, and differentiation capacity determine the bioactivity and new bone formation degree at the interface between the composite materials and the biological environment [48,49]. These phenomena require biomaterials with a hydrophilic surface (defined by low contact angle values <90°) to favor the adsorption of molecules from the biological fluids [15]. In this regard, one of the PLA deficiencies for the biomedical field refers to its inherent surface hydrophobicity that constantly imposes the incorporation or embedding of other materials to improve the wettability behavior [20,29,30].

**Figure 7:**
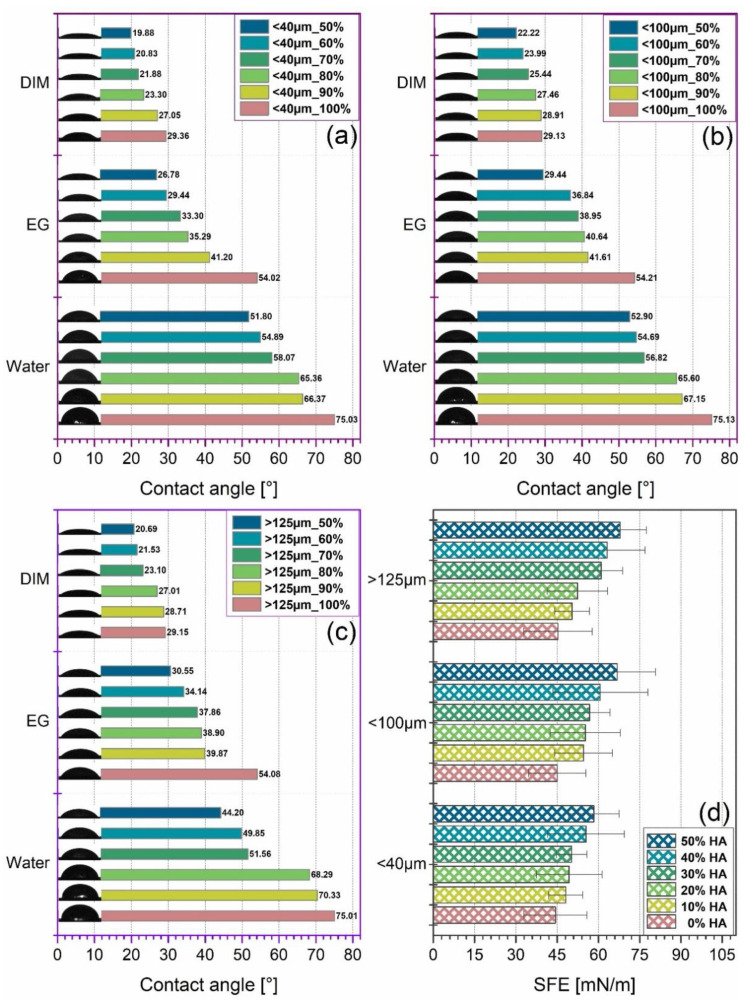
Surface wettability by contact angle measurements (three wetting agents: water, ethylene glycol, and diiodomethane) for the extruded filaments obtained from PLA and three-dimensional sorts of HA powder: (**a**) <40 μm, (**b**) <100 μm, and (**c**) >125 μm, each with 0–50% HA ratio, and (**d**) Surface Free Energy (SFE) results (OWRK method).

In this study, the surface hydrophilicity was evaluated by contact angle (CA) measurements with three different wetting agents/testing liquids (water, diiodomethane (DIM), and ethylene glycol (EG)) on the cast mixtures pellets. Further, the SFE was computed through the OWRK method [50] using the contact angle values previously measured (Figure 7). The surface free energy indicates the sum of dispersive and polar interactions recorded at the solid–liquid interface [51]. The nature of each testing liquid and its individual dispersive and polar component are known and reported [50,52]. For a good experimental set, the water and EG were used as polar liquids, while DIM was used as nonpolar/dispersive liquid.

It was noticed that the CA values formed a constant downward trendline once the incorporated HA ratio was increased from 10% to 50%, independent of the testing liquid or the HA particles sizes. The CA for water, DIM, and EG varied from ~66 to 20°, ~67 to 22°, and ~70 to 21° for HA particles in the <40 μm, <100 μm, and >125 μm ranges, respectively. Thus, all results revealed a significantly improved hydrophilic character (CA < 90°) of the composite materials as compared to the PLA alone. As expected, this is an immediate consequence of the surface morphology, a key factor for CA evolution (Figure 2, Figure 3 and Figure 4), correspondingly to the transition from small to larger particles and to rougher surfaces formation at >20–30% HA ratio (lower CA) [21,40,52]. Even though the incorporation of smaller particles (<40 μm) led to smoother surfaces, their surface microporosity and regular distribution (Figure 2) along with the decreased CA values, as compared to those of PLA, attest their beneficial role for improving the wettability features of the samples.

The inferred evolution of SFE values, also presented in Figure 7, revealed an upward tendency with the HA ratio increment, for all HA dimensional ranges. A high SFE value is a corresponding indicator of a pronounced wettability, both required in the additive manufacturing field in order to support the printing of continuous lines without the presence of the merging phenomenon [53]. Moreover, the higher the SFE, the higher the chances are for enhanced protein adsorption and overall cellular response for future implantable products [21,54,55,56].

### 3.4. Micro-CT Reconstruction

The micro-CT reconstruction (3D X-ray imaging) process requires the compilation of several hundred/thousands of 2D slices and volumetric data conversion into 3D images [57]. Specifically, this nondestructive technique allows the visualization and investigation of various sample types and afferent features from the surface to the entire volume [24,58,59,60].

**Figure 8:**
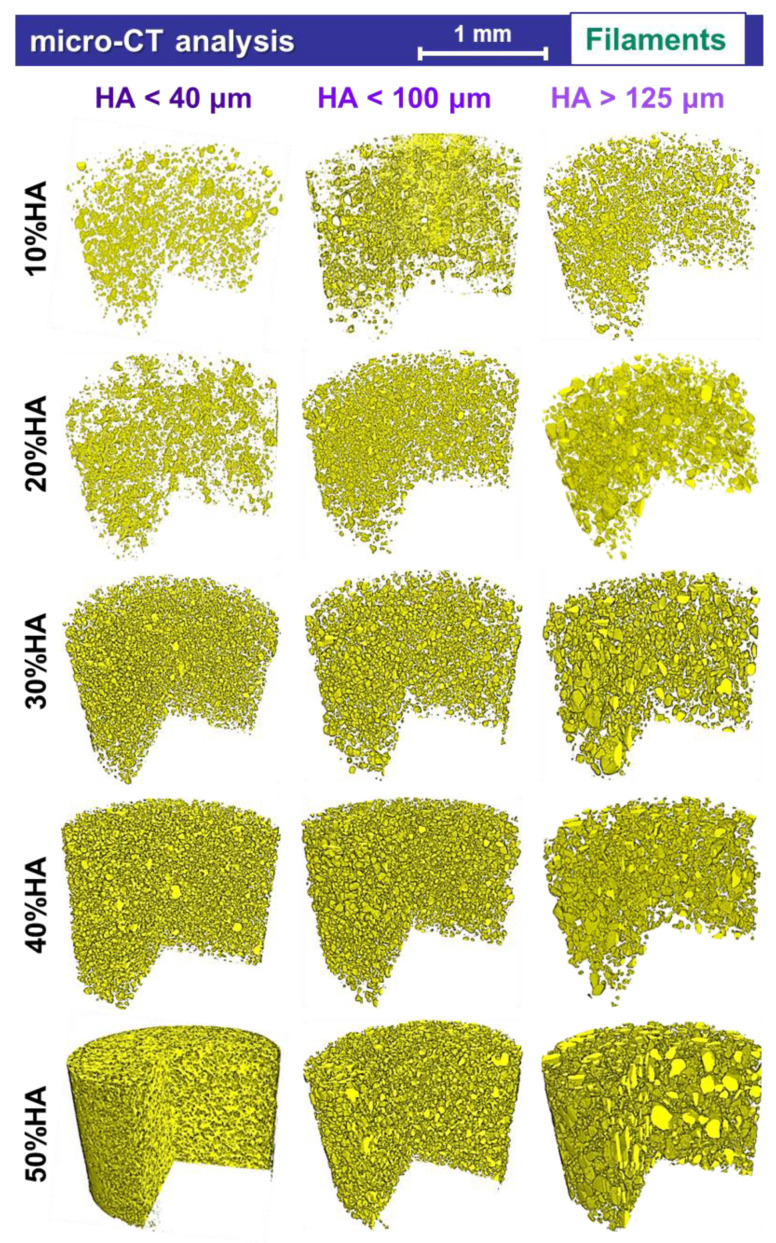
Micro-CT tridimensional reconstruction of the extruded filaments obtained from PLA and three-dimensional sorts of HA powder with 10–50% ratio.

In this study, the 3D reconstructions of all composite filaments provided a complete overview of the internal arrangement of the ceramic particles into the PLA matrix, as presented in Figure 8. Moreover, based on the CT technique ability to correlate the material type with their corresponding atomic number, an adequate setup of the software parameters rendered the possibility to display the areas of interest because of the two materials involved [61]. Thus, the blank spots, seen as voids, for all samples represent the actual polymeric matrix enclosing the micrometric HA particles (colored in yellow). The illustration of the entire volumes of the selected sections, minus a 3D triangular slice of each, was intentionally chosen in order to enhance the visualization of the particles distribution inside the filaments.

The HA particle-by-particle dispersion phenomenon evidenced in the cross-sectional surface view of the filaments (SEM micrographs from Figure 2, Figure 3 and Figure 4) was preserved and extended to the entire volume (Figure 8), independent of the ceramic particle size. The incremented incorporation of HA ratios radically reduced the free space between particles, that is, the polymeric matrix, while retaining the surface–volume homogeneity degree. Hence, once the HA particles size increased concomitant with the incorporated ratio, their uniform distribution led to the complex, organized, and gradual formation of internal structures evolved according to the filaments shape and size—even in height and diameter. In addition, no aggregates development could be depicted on either the samples surface or in their volume. Even more so, the individual outline of each particle can be observed in the 3D triangular sliced area of all samples, and distinctively emphasized for samples prepared with HA particles in the >125 μm range. Moreover, the rounding effect of the particles edges during extrusion is also confirmed throughout the volume of the filaments, which is also in good agreement with the morphological assessment.

Hence, the 3D reconstructions established that composite filaments with constant diameter can be achieved by both modulating the HA particles size and ratio. By targeting a homogenous surface–volume distribution of the ceramic particles in the polymeric matrix, a graded ceramic network can be induced inside the filaments. As such, the printing nozzle size and printing velocity could be modulated because of the filament type—in this case, the size and ratio of the incorporated HA particles [41].

### 3.5. Mechanical Features Evaluation

**Figure 9:**
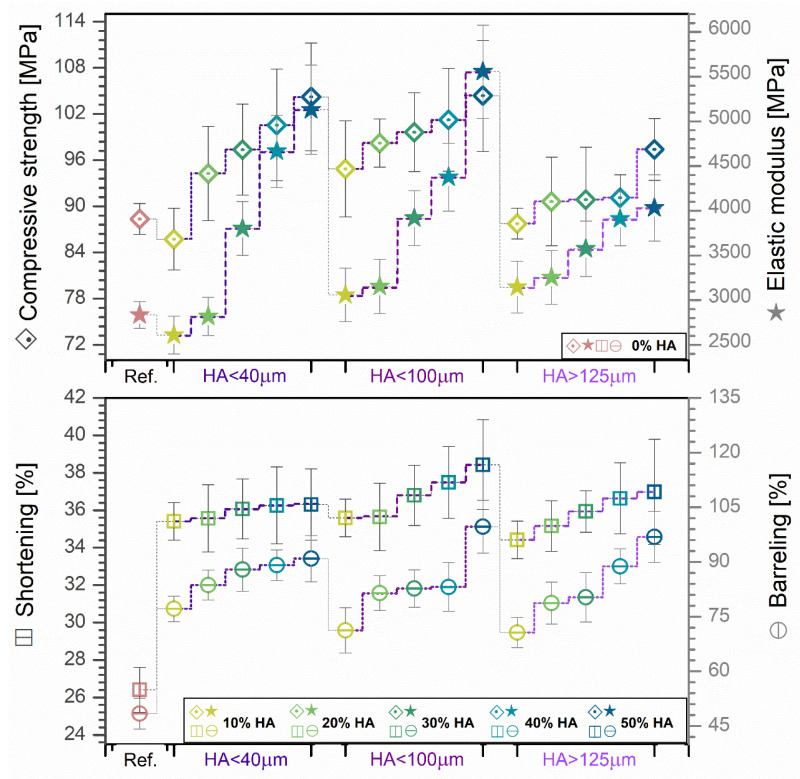
Variation in compressive strength and deformation degree (shortening and barreling) for the cast mixtures pellets obtained from PLA and three-dimensional sorts of HA powder with 0–50% ratio.

To support the preliminary investigations performed in this study, the mechanical behavior of all cast mixtures pellets was evaluated by compression testing. The afferent mean values and standard deviations of compressive strength and elastic modulus are comparatively exposed in Figure 9. The deformation degree by height and diameter (i.e., shortening and barreling degree) were computed by measuring the pellets before and after the compression was applied, and the graphical results are also included in Figure 9.

The variation in compressive strength and elastic modulus, along with the shortening and barreling degree, outlined similar linear ascending trends with the increase in the incorporated HA ratio, independent of the ceramic particles size. As compared to pellets obtained from PLA alone, both compressive strength and elastic modulus revealed enhanced values by ~1–1.18 (<40 μm), ~1.1–1.19 (<100 μm), ~1–1.10 (>125 μm), ~1–1.81 (<40 μm), ~1.1–1.96 (<100 μm), and ~1.1–1.43 (>125 μm), proving that the naturally derived HA particles indeed acted as reinforcement material for the PLA matrix. Another argument in this direction relates to their ability to sustain correspondingly higher deformation degrees than PLA before cracking, recorded with an augmentation factor of ~1.34–1.38 (<40 μm), ~1.35–1.46 (<100 μm), and ~1.31–1.40 (>125 μm) for shortening and ~1.60–1.88 (<40 μm), ~1.47–2.06 (<100 μm), and ~1.46–2 (>125 μm) for barreling.

The elastic modulus is also representative for the rigidity/stiffness degree [33], and hence, the gradual addition of HA particles led to more rigid composite materials, as it was expected and more importantly evidenced by the DSC findings (reduced crystallinity degree, Figure 6b). A certain amount of interaction between the ceramic particles and the polymer matrix is conceivable, as they connect in two different ways: physically because of the high porosity of the particles, which increases surface tension, and chemically based on the interactions between the polar groups of PLA and HA [33]. Both elements, along with the (decreased) crystallinity degree, enhance the adhesion between the polymeric and ceramic materials, increasing the stiffness of the produced composites as a result, even at low HA concentration, regardless of the particle size [62]. Moreover, the lower values recorded for samples with HA particles >125 μm can be attributed to the decreased dispersion degree and the particles tendency toward aggregates formation (Figure 4). Given the presented results, it would be safe to assume that the addition of HA has a large influence upon the enhancement of the elastic modulus of the composite materials, while an extensive particle size range tends to decrease it.

It has been reported that, for a good mechanical performance, the interactions between the ceramic particles and PLA matrix should occur because of three influencing factors: (1) the uniform surface–volume dispersion of the particles into the polymer [29] (demonstrated by both surface and volume evaluations—Figure 1, Figure 2, Figure 3 and Figure 4); (2) the physical adhesion and bonding strength at the ceramic–polymer interface due to the porous aspect of the particles facets, without generating pores [24,29,33] (checked in Figure 2, Figure 3, Figure 4 and Figure 8); (3) the chemical interplay between the polar groups of both materials, leading to elevated surface tension values [33] (Figure 7).

In the case of naturally derived HA incorporation, at different ratios and with variable particles sizes, into the PLA matrix, all three prerequisites were fulfilled and confirmed throughout the preliminary investigation program. All obtained results for the mechanical features are adequate for biomedical applications given that they far exceed the lower limit of resistance for the cortical bone, but not the upper one [11,12,20,63,64].

## 4. Conclusions

The feasibility to extrude natural composite HA/PLA filaments through the proposed preparation method was confirmed. The influence of concomitant modulation of the size, the ratio, and the effect of shape of the HA particles on the morphology, wettability, surface free energy, internal architecture, and mechanical behavior of the fabricated cast mixtures and filaments were surveyed for the first time.

All modifications performed for the ceramic component influenced directly the final features of the composite filaments. In this regard, a homogenous dispersion degree was outlined for all samples independent of the HA particles size or ratio, by complementary morphological investigations—for filaments surface/cross-section, and micro-CT—for the internal distribution and arrangement of the particles into the polymeric matrix. However, in order to preserve an invariable diameter and a smooth and uniform surface of the filaments, the HA ratio should be limited at a maximum of 40% and 20% only for particles in the <100 μm and >125 μm ranges, respectively.

In terms of adhesion at the HA/PLA interface, the particular polyhedral shape and microporous texture of the natural ceramic particles surface played a significant role for establishing a strong connection between the two components. Moreover, the absence of any outline porosity at the ceramic particle–polymer interface was also clearly observed. Combined with the overall enhanced mechanical features, one can notice that the HA component advantageously acted as mechanical reinforcement for the polymeric material, leading to values of the compressive strength and elastic modulus/stiffness degree in the bone-compatible limits. Further, the hydrophilic character, attained for all composite samples, exposed the HA ability to induce beneficial surface modifications of the polymeric matrix toward an improved wettability and favorable response of future cells implicitly, independent of the ceramic particles size or ratio.

Overall, the proposed technological route enables the adequate extrusion of natural composite filaments as feedstocks for future additive manufacturing scaffolding destined for bone clinical applications.

## Data Availability

Not applicable.

## Supplementary Materials

The following supporting information can be downloaded at: https://www.mdpi.com/article/10.3390/jfb13040199/s1, Figure S1 Morphological characterization of the three dimensional sorts of bovine bonederived HA powder. The dimensional analysis of the ceramic particles was performed with the ImageJ software. Scale bar: 200 μm
