# Supplementary material for: Influence of Ceramic Particles Size and Ratio on Surface—Volume Features of the Naturally Derived HA-Reinforced Filaments for Biomedical Applications
Source: J Funct Biomater. 2022 Oct 21;13(4):199. doi: 10.3390/jfb13040199 (PMC9590078; doi:10.3390/jfb13040199)
Supplement: Supplementary file 1 [file jfb-13-00199-s001.zip › jfb-1979234-supplementary.pdf]

Supplementary materials

Influence of Ceramic Particles Size and Ratio on Surface–Volume Features of the Naturally Derived HA-Reinforced Filaments for Biomedical Applications

Aura-Cătălina Mocanu [1], Florin Miculescu [1,*], Cătălina-Andreea Dascălu [1], Ştefan Ioan Voicu [2], Mădălina-Andreea Pandele [2,3], Robert-Cătălin Ciocoiu [1], Dan Batalu [1], Sorina Dondea [1], Valentina Mitran [4] and Lucian-Toma Ciocan [5]

[1] Department of Metallic Materials Science, Physical Metallurgy, Faculty of Materials Science and Engineering, University Politehnica of Bucharest, 313 Splaiul Independentei, J Building, 060042 Bucharest, Romania
[2] Department of Analytical Chemistry and Environmental Engineering, Faculty of Applied Chemistry and Materials Science, University Politehnica of Bucharest, 1-7 Gh. Polizu Str., 011061 Bucharest, Romania
[3] Advanced Polymer Materials Group, University Politehnica of Bucharest, 1-7 Gh. Polizu Str., 011061 Bucharest, Romania
[4] Department of Biochemistry and Molecular Biology, University of Bucharest, 91-95 Spl. Independentei, 050095 Bucharest, Romania
[5] Prosthetics Technology and Dental Materials Department, "Carol Davila" University of Medicine and Pharmacy, 37 Dionisie Lupu Street., 020022 Bucharest, Romania
* Correspondence: f_miculescu@yahoo.com or florin.miculescu@upb.ro; Tel.: +40-021-316-95-63

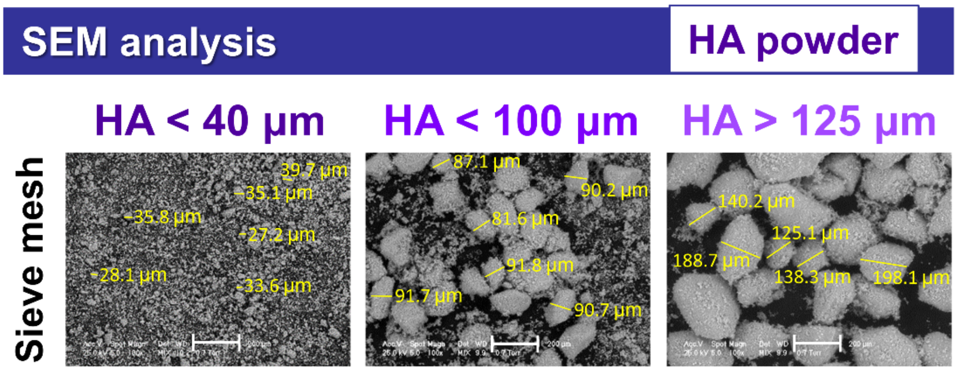

004.021.316.95.63

Figure S1. Morphological characterization of the three dimensional sorts of bovine bone-derived HA powder. The dimensional analysis of the ceramic particles was performed with the ImageJ software. Scale bar: 200 µm.
